# Supplementary material for: HIVprotI: an integrated web based platform for prediction and design of HIV proteins inhibitors
Source: J Cheminform. 2018 Mar 9;10:12. doi: 10.1186/s13321-018-0266-y (PMC5845081; doi:10.1186/s13321-018-0266-y)
Supplement: Supplementary file 2 — Additional file 2. Source code of HIVProtI web server. [file 13321_2018_266_MOESM2_ESM.zip › HIVprotI_Source-code/hivprotI/weka-3-6-6/doc/weka/clusterers/package-tree.html]

weka.clusterers Class Hierarchy


---


|  |  |  |
| --- | --- | --- |
| **Overview** \| **Package** \| Class \| **Tree** \| **Deprecated** \| **Index** \| **Help** \| **Weka's home** | |  |
| **PREV**   **NEXT** | **FRAMES**    **NO FRAMES**     **All Classes** |  |


---


## Hierarchy For Package weka.clusterers

**Package Hierarchies:**: All Packages

---

## Class Hierarchy

- java.lang.Object
  - weka.clusterers.**AbstractClusterer** (implements weka.core.CapabilitiesHandler, java.lang.Cloneable, weka.clusterers.Clusterer, weka.core.RevisionHandler, java.io.Serializable)
    - weka.clusterers.**AbstractDensityBasedClusterer** (implements weka.clusterers.DensityBasedClusterer)
      - weka.clusterers.**MakeDensityBasedClusterer** (implements weka.clusterers.NumberOfClustersRequestable, weka.core.OptionHandler, weka.core.WeightedInstancesHandler)- weka.clusterers.**RandomizableDensityBasedClusterer** (implements weka.core.OptionHandler, weka.core.Randomizable)
          - weka.clusterers.**EM** (implements weka.clusterers.NumberOfClustersRequestable, weka.core.WeightedInstancesHandler)
      - weka.clusterers.**CLOPE** (implements weka.core.OptionHandler, weka.core.TechnicalInformationHandler)- weka.clusterers.**DBScan** (implements weka.core.OptionHandler, weka.core.TechnicalInformationHandler)- weka.clusterers.**HierarchicalClusterer** (implements weka.core.CapabilitiesHandler, weka.core.Drawable, weka.core.OptionHandler)- weka.clusterers.**OPTICS** (implements weka.core.OptionHandler, weka.core.TechnicalInformationHandler)- weka.clusterers.**RandomizableClusterer** (implements weka.core.OptionHandler, weka.core.Randomizable)
                - weka.clusterers.**Cobweb** (implements weka.core.Drawable, weka.core.TechnicalInformationHandler, weka.clusterers.UpdateableClusterer)- weka.clusterers.**FarthestFirst** (implements weka.core.TechnicalInformationHandler)- weka.clusterers.**sIB** (implements weka.core.TechnicalInformationHandler)- weka.clusterers.**SimpleKMeans** (implements weka.clusterers.NumberOfClustersRequestable, weka.core.WeightedInstancesHandler)- weka.clusterers.**XMeans** (implements weka.core.TechnicalInformationHandler)
                - weka.clusterers.**RandomizableSingleClustererEnhancer** (implements weka.core.OptionHandler, weka.core.Randomizable)- weka.clusterers.**SingleClustererEnhancer** (implements weka.core.OptionHandler)
                    - weka.clusterers.**FilteredClusterer**
    - weka.core.**Check** (implements weka.core.OptionHandler, weka.core.RevisionHandler)
      - weka.core.**CheckScheme**
        - weka.clusterers.**CheckClusterer**
      - weka.clusterers.**ClusterEvaluation** (implements weka.core.RevisionHandler, java.io.Serializable)

## Interface Hierarchy

- weka.clusterers.**Clusterer**
  - weka.clusterers.**DensityBasedClusterer**
  - weka.clusterers.**NumberOfClustersRequestable**- weka.clusterers.**UpdateableClusterer**

---


|  |  |  |
| --- | --- | --- |
| **Overview** \| **Package** \| Class \| **Tree** \| **Deprecated** \| **Index** \| **Help** \| **Weka's home** | |  |
| **PREV**   **NEXT** | **FRAMES**    **NO FRAMES**     **All Classes** |  |


---
